# Pleurochrysome: A Web Database of *Pleurochrysis* Transcripts and Orthologs Among Heterogeneous Algae

**DOI:** 10.1093/pcp/pcv195

**Published:** 2016-01-07

**Authors:** Naoki Yamamoto, Toru Kudo, Shoko Fujiwara, Yukiko Takatsuka, Yasutaka Hirokawa, Mikio Tsuzuki, Tomoyuki Takano, Masaaki Kobayashi, Kunihiro Suda, Erika Asamizu, Koji Yokoyama, Daisuke Shibata, Satoshi Tabata, Kentaro Yano

**Affiliations:** ^1^Bioinformatics Laboratory, School of Agriculture, Meiji University, 1-1-1 Higashi-mita, Tama-ku, Kawasaki, Kanagawa, 214-8571 Japan; ^2^School of Life Sciences, Tokyo University of Pharmacy and Life Sciences, 1432-1 Horinouchi, Hachioji, Tokyo, 192-0392 Japan, CREST, Japan; ^3^Kazusa DNA Research Institute, 2-6-7 Kazusa-kamatari, Kisarazu, Chiba, 292-0818 Japan; ^4^These authors contributed equally to this work.; ^5^Present address: International Rice Research Institute, DAPO 7777, Metro Manila 1301, Philippines.

**Keywords:** Coccolithophorids, Expressed sequence tag, Haptophyta, Integrated database, Ortholog, *Pleurochrysis*

## Abstract

*Pleurochrysis* is a coccolithophorid genus, which belongs to the Coccolithales in the Haptophyta. The genus has been used extensively for biological research, together with *Emiliania* in the Isochrysidales, to understand distinctive features between the two coccolithophorid-including orders. However, molecular biological research on *Pleurochrysis* such as elucidation of the molecular mechanism behind coccolith formation has not made great progress at least in part because of lack of comprehensive gene information. To provide such information to the research community, we built an open web database, the Pleurochrysome (http://bioinf.mind.meiji.ac.jp/phapt/), which currently stores 9,023 unique gene sequences (designated as UNIGENEs) assembled from expressed sequence tag sequences of *P. haptonemofera* as core information. The UNIGENEs were annotated with gene sequences sharing significant homology, conserved domains, Gene Ontology, KEGG Orthology, predicted subcellular localization, open reading frames and orthologous relationship with genes of 10 other algal species, a cyanobacterium and the yeast *Saccharomyces cerevisiae.* This sequence and annotation information can be easily accessed via several search functions. Besides fundamental functions such as BLAST and keyword searches, this database also offers search functions to explore orthologous genes in the 12 organisms and to seek novel genes. The Pleurochrysome will promote molecular biological and phylogenetic research on coccolithophorids and other haptophytes by helping scientists mine data from the primary transcriptome of *P. haptonemofera.*

## Introduction

*Pleurochrysis* are one of the coccolithophorids, which produce calcified scales, called coccoliths, on the cell surface. Coccolithophorids are included in two orders of the Haptophyta, the Coccolithales and Isochrysidales (Edvardsen et al. 2000). As representatives of the orders, *Pleurochrysis* (Coccolithales) and *Emiliania* (Isochrysidales) have been used for coccolithophorid research as they can be cultured in the laboratory (Berges et al. 2001, Marsh 2003). To date, research with the two genera has revealed that the coccolith production mechanism and other morpho-physiological characteristics are very different between the two orders, e.g. the subcellular compartments where coccolith production occurs (van der Wal et al. 1983, Inouye and Pienaar 1984, Westbroek et al. 1989), chemical variation of coccolith acid polysaccharides (van Emburg et al. 1986, Marsh et al. 1992, Hirokawa et al. 2005) and the morphology of the base plate of the coccolith (de Vrind-de Jong and de Vrind 1997). Furthermore, although the Haptophyta are known as one of the old-fashioned supergroup Chromalveolates which probably gained photosynthetic ability through secondary endosymbiosis with rhodophyte(s) (Jordan and Chamberlain 1997, Cavalier-Smith 1999), the phylogenetic position of the Haptophyta is still being uncovered (Burki et al. 2007, Hackett et al. 2007, Burki et al. 2012).

To understand the molecular mechanisms behind the biological functions and to uncover the evolutionary history, comprehensive gene information is very important since such information accelerates molecular biological studies and enables comparative genomics. Recently, a reference genome of *Emiliania huxleyi*, which is the predominant coccolithophorid species in the current ecosystem, has been published (Read et al. 2013). On this *E. huxleyi* genome, 30,569 gene models were predicted (Read et al. 2013) and their sequence information is available in the genome portal of the Joint Genome Institute (JGI) (Nordberg et al. 2014). However, there is no *Pleurochrysis* database, and publicly available gene sequence information on *Pleurochrysis* is limited to only 156 nucleotide and 64 protein sequences deposited in the GenBank database (Benson et al. 2013). Therefore, to facilitate research with *Pleurochrysis* toward elucidating phycological issues including the coccolith formation mechanism and the evolutionary history, establishing its comprehensive gene information and having a freely available and easily accessible platform to provide the information, more specifically a public web database, are crucial.

Here, we introduce a *Pleurochrysis* transcript sequence database, the Pleurochrysome (http://bioinf.mind.meiji.ac.jp/phapt/). The Pleurochrysome stores >9,000 transcript sequences of *Pleurochrysis haptonemofera* which were constructed from expressed sequence tag (EST) sequences newly or previously analyzed (Fujiwara et al. 2007) with their structural and functional annotation derived from our bioinformatic analyses. To allow comparative genomics approaches, the database incorporates information of orthologous sequences among 12 divergent unicellular species: 10 algae, a cyanobacterium and a yeast. The database offers fundamental and unique search functions to explore candidate genes relevant to targeted traits and novel genes in *Pleurochrysis.* To our knowledge, this is the first ‘full-blown’ web database which provides comprehensive transcript information in the Haptophyta.

## Results

### Information stored in the database

To obtain comprehensive transcript information, we newly analyzed 4,924 EST sequences of *P. haptonemofera* in addition to the 9,564 ESTs which we had already sequenced (Fujiwara et al. 2007; DDBJ accession Nos. HX954614–HX969076). These EST sequences were assembled into contigs after quality control. The resulting sequences of 1,868 contigs and 7,155 singlets, together designated as ‘UNIGENEs’, were stored in the Pleurochrysome (http://bioinf.mind.meiji.ac.jp/phapt/) with the original EST information. To provide a platform for similarity searches against other algae, this database also stores protein sequences collected from public databases for 10 algal species, *Chlamydomonas reinhardtii*, *Cyanidioschyzon merolae*, *Ectocarpus siliculosus*, *Emiliania huxleyi*, *Guillardia theta* (nuclear and nucleomorph), *Hemiselmis andersenii* (nucleomorph), *Micromonas* sp. RCC299, *Ostreococcus lucimarinus* CCE9901, *Phaeodactylum tricornutum* and *Thalassiosira pseudonana*, as well as *Synechocystis* sp. PCC6803 (a cyanobacterium) representing the likely origin of primary plastids, and *Saccharomyces cerevisiae* (yeast) as a non-algal outgroup (Supplementary Table S1).

The *P. haptonemofera* UNIGENEs were functionally annotated based on analyses using programs such as BLAST (Camacho et al. 2008), InterProScan (Quevillon et al. 2005) and the KEGG Automatic Annotation Server (Mao et al. 2005). BLAST searches were performed against the National Center for Biotechnology Information (NCBI) nucleotide database (nt), EST database (dbEST) and protein database (nr) using each UNIGENE sequence as a query. Applying 1E-10 as a threshold e-value, 3,215 UNIGENEs were annotated with at least one similar sequence in the databases. Conserved domain information and Gene Ontology (GO) information were retrieved by InterProScan. Conserved domains were detected in 5,786 UNIGENEs, and GO terms were assigned to 3,157 of the UNIGENEs where a domain was found. Analysis with the KEGG Automatic Annotation Server yielded KEGG Orthology information for 994 UNIGENEs. To obtain information related to subcellular localization of polypeptides potentially encoded by UNIGENEs, analyses using the SignalP (Petersen et al. 2011), ChloroP (Emanuelsson et al. 1999) and WoLF PSORT (Horton et al. 2007) programs were also performed. SignalP predicted ‘Signal peptide’ and ‘Signal anchor’ in 3,912 and 6,771 UNIGENEs, respectively; ChloroP predicted that 7,485 UNIGENEs may encode plastid-localized proteins. It should be noted that, when looking at this information, the results of open reading frame (ORF) estimation should also be referred to since the probability of the frame being the true ORF of the transcript is not considered in prediction of localization. The estimation of ORFs was performed using the ESTScan tool (Iseli et al. 1999). The result showed that significant ORFs were deduced in 4,210 UNIGENEs. Hence, the subcellular localization predicted in the estimated ORFs of the 4,210 UNIGENEs would be more reliable than that in the other ORFs. Orthologous genes for UNIGENEs were predicted based on results obtained by analysis with OrthoMCL (Chen et al. 2006) and BLAST search as described later. These annotation and prediction results are collectively housed in the Pleurochrysome.

### Construction and search functions of the database

The Pleurochrysome was developed as a web database so that users can access the database via the Internet using a web browser. The database and user interfaces were built as a typical server–client system using open source software: Linux (CentOS release 5.10, 64-bit) as the operating system; Apache HTTP server (version 2.2.27) as the Web server; MySQL (version 5.0.95) as the relational database management system; and PHP (version 5.3.3) as the server-side scripting language. Any user can freely access all the functions and data without signing in.

**Fig. 1:**
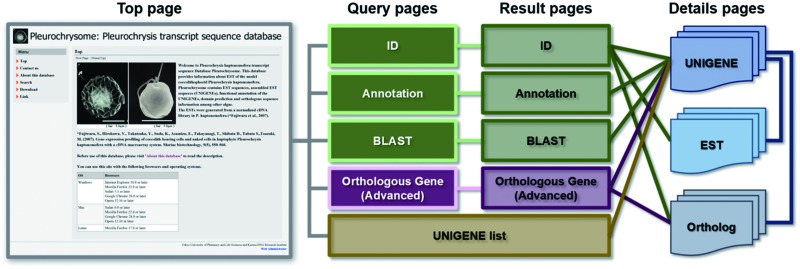
Functional relationship among pages comprising the Pleurochrysome web site. From left to right, the top page provides hyperlinks to query pages where an ID search, BLAST search, keyword search against annotation information, or an orthologous gene search can be submitted, and to the UNIGENE list page. After submitting a search, unless no hit is found, the corresponding result pages will appear. The result pages and the UNIGENE list page display lists of UNIGENE, EST and/or orthologous genes found in other organisms and provides hyperlinks to the details page describing extensive information on UNIGENEs, ESTs or orthologous genes (Ortholog). Each UNIGENE is assigned with ESTs which were assembled for the UNIGENE and with the orthologous genes.

**Fig. 2:**
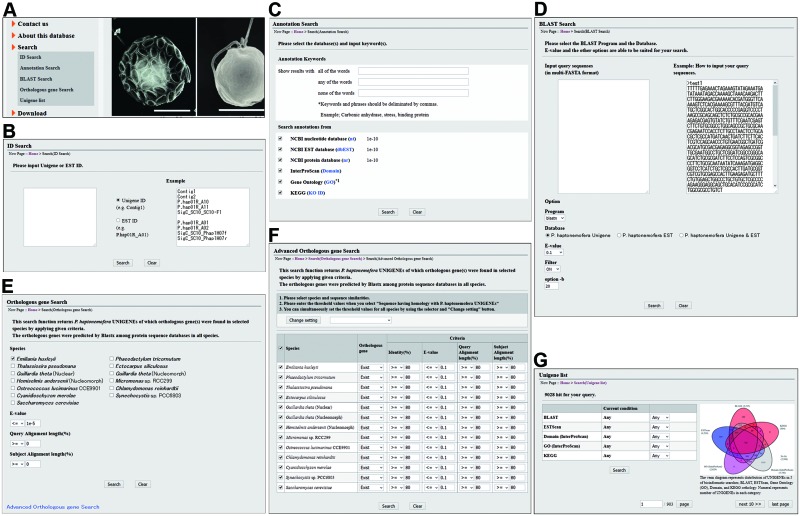
Search query pages in the Pleurochrysome. Under ‘Search’ in the menu on the upper left of pages (A), there are hyperlinks to search query pages, namely ID Search (B), Annotation Search (C), BLAST Search (D) and Ortholougs Gene Search (E), and a UNIGENE list (G) page. A hyperlink to the Advanced Orthologous Gene Search (F) is on the lower left of the Orthologous Gene Search page.

To search information stored in this database, ‘Orthologous Gene Search’ and ‘UNIGENE list’ functions have been implemented in the Pleurochrysome in addition to fundamental search functions including ‘ID Search’, ‘Annotation Search’ and ‘BLAST Search’ (Figs. 1, 2). These search functions are accessible from the top page (Figs. 1, 2A). All search functions eventually lead users to a ‘UNIGENE Details’, ‘EST Details’ or ‘Ortholog Details’ page via the search result pages (Fig. 1). In addition to links to the search functions, the top page also has links to contact information, including the E-mail address for those to whom correspondence should be addressed, and basic information related to the Pleurochrysome such as data sources, bulk download and related external links. Detailed explanations and instructions for each function and page will be described in the following sections.

### Fundamental search functions

**Fig. 3:**
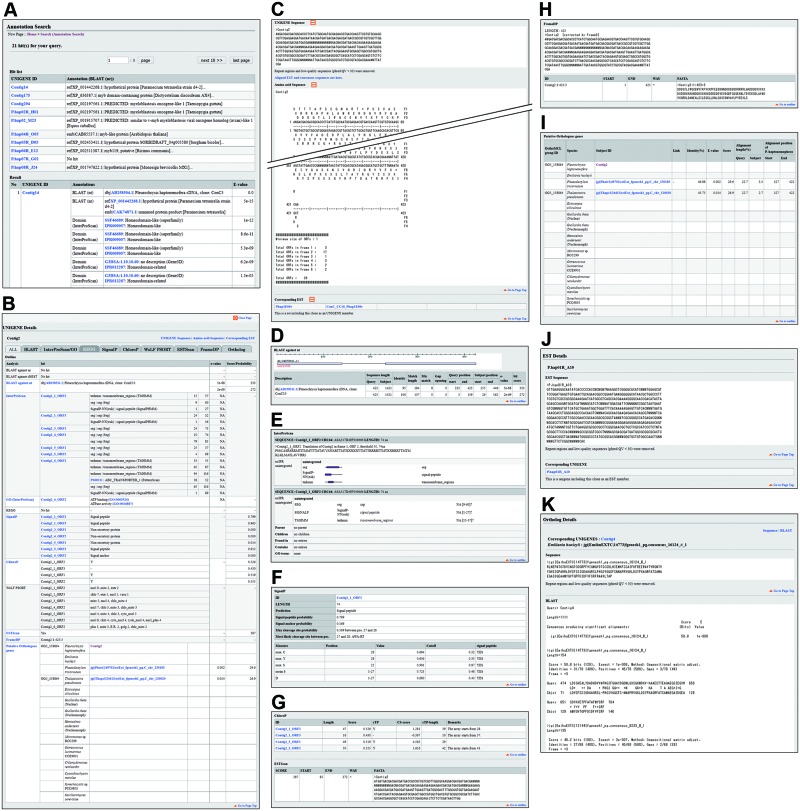
Search result pages and sequence detail pages in the Pleurochrysome. The search result page is essentially comprised of a page selector, a ‘Hit list’ table which is a simple list of hit sequences, and a ‘Result’ table showing more detailed results in all search functions, though a result page for an Annotation search is presented here (A). The ‘UNIGENE Details’ is a vertically long page showing a table summarizing the results of annotation analyses of the UNIGENE (B); nucleotide and deduced amino acid sequences of the UNIGENE, and corresponding EST sequence IDs (C); results of a BLAST search against NCBI nucleotide, protein and EST databases (D); results of a search for conserved domain and Gene Ontology terms by InterProScan (E); results of signal peptide prediction by SignalP (F); results of prediction of chloroplast localization by ChloroP (G); results of open reading frame prediction by FrameDB (H); and a list of putative orthologous genes found in various algal species, a cyanobacterial species and a yeast species (I). The tabs located at the top of the ‘UNIGENE Details’ page work to specify information to be shown in this page (B). An ‘EST Details’ page shows the nucleotide sequence of the EST and UNIGENE ID where the EST was assembled (J). An ‘Ortholog Details’ page shows the amino acid sequence encoded by a gene which is listed as a putative orthologous gene of UNIGENEs and the BLASTX result between the putative ortholog and the UNIGENE (K).

The ‘ID Search’ function provides the simplest route to reach the ‘UNIGENE Details’ and ‘EST Details’ pages when a user has the identifier (ID) of a UNIGENE or EST of interest. In this search, IDs used in this database for *P. haptonemofera* UNIGENEs and ESTs are accepted as queries (Fig. 2B). In this search function, a single ID can be accepted in each line of the text box to input queries. Since there exist pairs of a UNIGENE and an EST sharing the same ID, users need to specify which kind of ID, the UNIGENE or EST, is being used as a query. After clicking on the ‘Search’ button to execute the search, unless no hit is found, the result page will appear displaying two tables: the ‘Hit list’ table and the ‘Result’ table. The ‘Hit list’ table represents a compact list of UNIGENE/EST IDs matched with the queries and simple annotation obtained from the BLAST search against the nr database. Each UNIGENE/EST ID on this table provides a link to jump to the UNIGENE/EST in the ‘Result’ table. This table represents the UNIGENE/EST IDs with more detailed annotation obtained not only from the BLAST search against the nr database but also from other searches such as domain searches with InterProScan. Each UNIGENE/EST ID in the ‘Result’ table provides a link to open a ‘UNIGENE Details’ or ‘EST Details’ page. The results of any searches in the Pleurochrysome are represented in essentially the same manner unless otherwise noted (Fig. 3A).

Every UNIGENE and EST has a unique ‘Details’ page. The ‘UNIGENE Details’ page shows all information related to the UNIGENE such as its sequence, the EST sequences comprising the UNIGENE, and detaild annotations (Fig. 3B–J). Although the page is vertically long, the ‘Outline’ table provides summarized information and a quick jump to detailed information (Fig. 3B). In addition, tabs shown just below the UNIGENE ID at the top specify the information to be shown (Fig. 3B). The ‘EST Details’ page shows the sequence of the EST and the UNIGENE ID associated with the EST, with a hyperlink to open the ‘UNIGENE Details’ page of the UNIGENE (Fig. 3J). The ‘Ortholog Details’ page provides a protein sequence of the 12 unicellular organisms and UNIGENE ID with which an orthologous relationship is predicted (Fig. 3K).

The ‘Annotation Search’ is a keyword search function to find UNIGENEs (Fig. 2C). The targeted information in this search is the results of searches with BLAST, InterProScan and the KEGG Automatic Annotation Server. The ‘BLAST Search’ page supports BLAST searches against *P. haptonemofera* UNIGENE and/or EST databases with blastn, tblastn and tblastx programs and against protein databases of the 10 algal species, the cyanobacterium and the yeast with blastp and blastx programs (Fig. 2D). Any query must be submitted in the FASTA format.

### Orthologous gene searches

Knowledge of the distribution of orthologous genes among related species is important to estimate gene function and to understand speciation. To provide such information on the Pleurochrysome, we investigated orthologous relationship among *P. haptonemofera* UNIGENEs and protein-coding genes of other species. Orthologous groups were first built among the 10 algal species, the cyanobacterium and the yeast, but without *P. haptonemofera*, by using OrthoMCL. Thereafter, *P. haptonemofera* UNIGENEs were connected with the orthologous groups based on the best hit counterpart in the result of a blastx search with the UNIGENE nucleotide sequences as a query against each protein database of the 12 species. The resulting information on the orthologous relationship is stored in this database, so that the user can explore orthologous gene candidates via the ‘Orthologous Gene Search’ function by searching within the orthologous information. To perform this search, users need to select the species, and also set the e-value threshold, and the alignment length of the query sequence and the similar sequence (subject) as criteria to judge whether the similarity is significant (Fig. 2E). The ‘Orthologous Gene Search’ function explore orthologous genes by four steps: (i) once executed, the search program seeks UNIGENEs having the best hit counterpart satisfying the thresholds in the selected species; (ii) when a satisfactory pair of a UNIGENE and the best hit counterpart is found, all members of the orthologous group to which the best hit counterpart belongs are retrieved; (ii) the member genes of the orthologous group are again filtered by the thresholds; and then (iv) the genes selected by the third step are retrieved as a result of the search. In the basic ‘Orthologous Gene Search’, a search by selecting multiple species will provide information on all UNIGENEs assigned with at least one orthologous gene in any species selected.

For a more rigorous search for orthologous genes, the ‘Advanced Orthologous Gene Search’ is available from a link located at the bottom left of the search and result pages of the ‘Orthologous Gene Search’ (Fig. 2E, F). In this advanced search, users can set the threshold (e-value, alignment length of the query sequence and the similar sequence, and identity) for each species. Also, users can precisely restrict the distribution of orthologous genes among the species by selecting ‘Exist’, ‘Not exist’ or ‘Any’ in the ‘Orthologous gene’ column. When a user selects ‘Exist’ or ‘Not exist’ for a species, setting the threshold to evaluate similarity and significance is required, as in the basic ‘Orthologous Gene Search’. To change option settings for multiple species at once, the user selects the species to which a change will be made by clicking a selection box, selecting an option from a pull-down menu, setting arbitrary conditions such as the thresholds, then clicking the ‘Change setting’ button. The search procedure is essentially the same as that for the basic ‘Orthologous Gene Search’. This search function can be used to explore UNIGENEs with a distribution that is correlated among species with respect to evolution, biological function or both. For instance, to explore candidates for a gene related to a common mechanism in coccolith formation between the coccolithophorids *P. haptonemofera* and *E. huxleyi*, the ‘Orthologous gene’ option should be set as ‘Exist’ only in *E. huxleyi* but as ‘Not exist’ in other species. By setting the other options the same among all the species (e-value ≤1E-10, query alignment length ≥60%, subject alignment length ≥60%, identity ≥40%), 162 UNIGENEs including putative carbonic anhydrase genes are found (Supplementary Fig. S1).

### UNIGENE list

‘UNIGENE list’ is a page simply to browse UNIGENEs with an accessory function to filter the UNIGENEs by results (hit or no hit) of searches using BLAST, KEGG, InterProScan (domain and GO) and ESTScan (Fig. 2G). Filtering can be performed by setting conditions in a table (Fig. 2G). For instance, when ‘Hit’ was selected for ‘Domain (InterProScan)’ and ‘No hit’ for other options, 1,515 UNIGENEs were retained. These UNIGENEs are potentially novel genes encoding a protein having a molecular function related to the known domain.

### Conclusion and future direction

Coccolithophorids have a great potential to contribute to maintenance of the ecology of the earth. To promote effective utilization of their remarkable functions and genetic resource, we constructed the Pleurochrysome on the basis of 9,023 *P. haptonemofera* UNIGENEs. In our analysis, comparison between the *P. haptonemofera* UNIGENEs and 39,126 gene models of *E. huxleyi* (Read et al. 2013) showed that only half of the UNIGENEs share significant similarity with *E. huxleyi* genes (data not shown). This result indicates that the UNIGENEs may include a lot of novel sequences, demonstrating the importance of the information provided in this database.

In the current version of the Pleurochrysome, whereas orthologous groups were generated using the OrthoMCL for the other 12 organisms, blastx was used to assign each UNIGENE with the orthologous groups. This is because many UNIGENEs are likely to be partial transcripts containing an incomplete coding sequence. Thus, to improve the accuracy of orthologous gene prediction, obtaining complete coding sequences of all *P. haptonemofera* genes is required. To that end, we are planning to increase the comprehensive gene information of *P. haptonemofera* using next-generation sequencing techniques such as whole-genome shotgun and RNA sequencing. By integrating the current EST sequences and the upcoming sequences, this database will be developed to provide more comprehensive sequences and more precise computational prediction of functional and structural annotation for *Pleurochrysis* genes, like other plant databases (Sakai et al. 2013, Ohyanagi et al. 2014, Aya et al. 2015, Bolser et al. 2015, Krishnakumar et al. 2015).

We welcome requests for updating the data and functions in the Pleurochrysome. Comments, suggestions and questions should be directed to Kentaro Yano at kyano@isc.meiji.ac.jp. The availability of particular cDNA clones of ESTs should be checked by sending an E-mail to Shoko Fujiwara at fujiwara@toyaku.ac.jp.

## Materials and Methods

### EST generation and UNIGENE construction

Growth conditions for *Pleurochrysis haptonemofera* (Inouye et Chihara) Gayral et Fresnel and methods for constructing a normalized cDNA library have been described previously (Fujiwara et al. 2007). Briefly, isolated coccolith-bearing cells (C-cells) and naked cells were stably maintained in ESM medium under continuous light with constant bubbling of air at 20°C. The cells were harvested before or after various treatments such as high and low salinity, high (30°C) and low (10°C) temperature, darkness, NaHCO_3_ supply (for C-cells) and low pH (pH 5.5, for C-cells). The absence of contamination of the culture with other microorganisms was confirmed by microscopy. Polyadenylated RNA was prepared from a mixture of cells treated under various conditions and used for construction of a normalized cDNA library according to previous studies (Bonaldo et al. 1996, Asamizu et al. 1999). In addition to the 9,564 terminal sequences of the library that have been published (Fujiwara et al. 2007), terminal sequence of another 4,924 cDNA clones were newly determined by Sanger sequencing. The resulting raw sequencing data of the 14,488 total clones were processed by Phred for base calling (Ewing and Green 1998). After masking vector sequences using the Crossmatch program (Ewing and Green 1998) and elimination of repeated and ambiguous sequences using in-house PERL scripts, the EST sequences were assembled into contigs and singlets using the CAP3 program with the default settings (Huang and Madan 1999).

### Homology search for UNIGENEs

For annotation of the UNIGENEs, the nucleotide database (nt), EST database (dbEST) and protein database (nr) of the NCBI were used in homology searches using the BLAST program (version 2.2.18) (Camacho et al. 2008). To prepare information on orthologous genes, the ‘Assign your proteins to OrthoMCL Groups’ function provided in the OrthoMCL DB (Chen et al. 2006; http://www.orthomcl.org/orthomcl/) and the BLAST program (version 2.2.31) were used. The analysis with the OrthoMCL was performed to build orthologous gene groups for the 12 selected model unicellular organisms: *Chlamydomonas reinhardtii*, *Cyanidioschyzon merolae*, *Ectocarpus siliculosus*, *Emiliania huxleyi*, *Guillardia theta* (nuclear and nucleomorph), *Hemiselmis andersenii* (nucleomorph), *Micromonas* sp. RCC299, *Ostreococcus lucimarinus* CCE9901, *Phaeodactylum tricornutum*, *Thalassiosira pseudonana*, *Synechocystis* sp. PCC6803 and *Saccharomyces cerevisiae.* The data sources for the protein sequences are summarized in Supplementary Table S1. The blastx searches with the BLAST program were performed with the protein database of each species separately, setting the ‘-dbsize’ option to 25,000 so that e-values are comparable among the species. Results obtained from both OrthoMCL and BLAST were stored as a relational database so that the relationship between each UNIGENE and the orthologous groups can be searched easily.

### Assignment of conserved domains, Gene Ontology and KEGG Orthology to UNIGENEs

Information on conserved domain and GO for UNIGENEs was obtained by analysis using the InterProScan tool (Quevillon et al. 2005) with default settings. To assign KEGG Orthology IDs (Mao et al. 2005), UNIGENEs were analyzed using the KEGG Automatic Annotation Server (Moriya et al. 2007). This search was conducted by the assignment method based on bi-directional best hits against the data of any species.

### Prediction of coding sequences in UNIGENEs

Coding sequences were predicted by a combined strategy. First, we identified 440 UNIGENEs containing a putative full-length protein-coding sequence by analysis with FrameDP (Gouzy et al. 2009) using SWISS-PROT (Boeckmann et al. 2003) as a reference. Using the 440 putative full-length protein-coding sequences as a training data set, a coding matrix was generated, and ORFs of UNIGENEs were predicted by the ESTScan program with the default settings (Iseli et al. 1999).

### Prediction of subcellular localization

Subcellular localization of proteins encoded by UNIGENEs was predicted using SignalP, ChloroP and WoLF PSORT with the default settings (Emanuelsson et al. 1999, Horton et al. 2007, Petersen et al. 2011). Translated protein sequences inferred by InterProScan were used in the prediction. In the ChloroP search, translated sequences were used after removing a signal peptide sequence detected at the N-terminus by the SignalP search.

### Availability of cDNA

EST information is available at http://bioinf.mind.meiji.ac.jp/phapt. The availability of particular cDNA clones should be checked by sending an E-mail to Shoko Fujiwara at fujiwara@toyaku.ac.jp.

## Funding

This work was supported in part by Grants-in-Aid for Scientific Research (Nos. 24113518 and 26113716 to K.Y. , and Nos. 20570059 and 24570114 to S.F.) from the Japan Society for Promotion of Science (JSPS); MEXT-Supported Program for the Strategic Research Foundation at Private Universities (2014-2018); Research Funding for Computational Software Supporting Program from Meiji University [Research Funding to K.Y.].

## Supplementary Material

Supplementary Data

## Abbreviations

C-cells: coccolith-bearing cells
EST: expressed sequence tag
GO: Gene Ontology
ID: identifier
ORF: open reading frame
