# Supplementary material for: Pleurochrysome: A Web Database of Pleurochrysis Transcripts and Orthologs Among Heterogeneous Algae
Source: Plant Cell Physiol. 2016 Jan 7;57(1):e6. doi: 10.1093/pcp/pcv195 (PMC4722176; doi:10.1093/pcp/pcv195)
Supplement: Supplementary Data [file supp_57_1_e6__index.html]

Pleurochrysome: A web-database of Pleurochrysis transcripts and orthologues among heterogeneous algae — Pleurochrysome: A Web Database of Pleurochrysis Transcripts and Orthologs Among Heterogeneous Algae — Pleurochrysome: A Web Database of Pleurochrysis Transcripts and Orthologs Among Heterogeneous Algae — Supplementary Data 

# Pleurochrysome: A Web Database of *Pleurochrysis* Transcripts and Orthologs Among Heterogeneous Algae

## Supplementary Data

files

- Supplementary Data - pdf file
- Supplementary Data - xlsx file
