# Supplementary material for: Pleurochrysome: A Web Database of Pleurochrysis Transcripts and Orthologs Among Heterogeneous Algae
Source: Plant Cell Physiol. 2016 Jan 7;57(1):e6. doi: 10.1093/pcp/pcv195 (PMC4722176; doi:10.1093/pcp/pcv195)
Supplement: Supplementary Data [file supp_pcv195_pcp-2015-e-00629-File005.pdf]

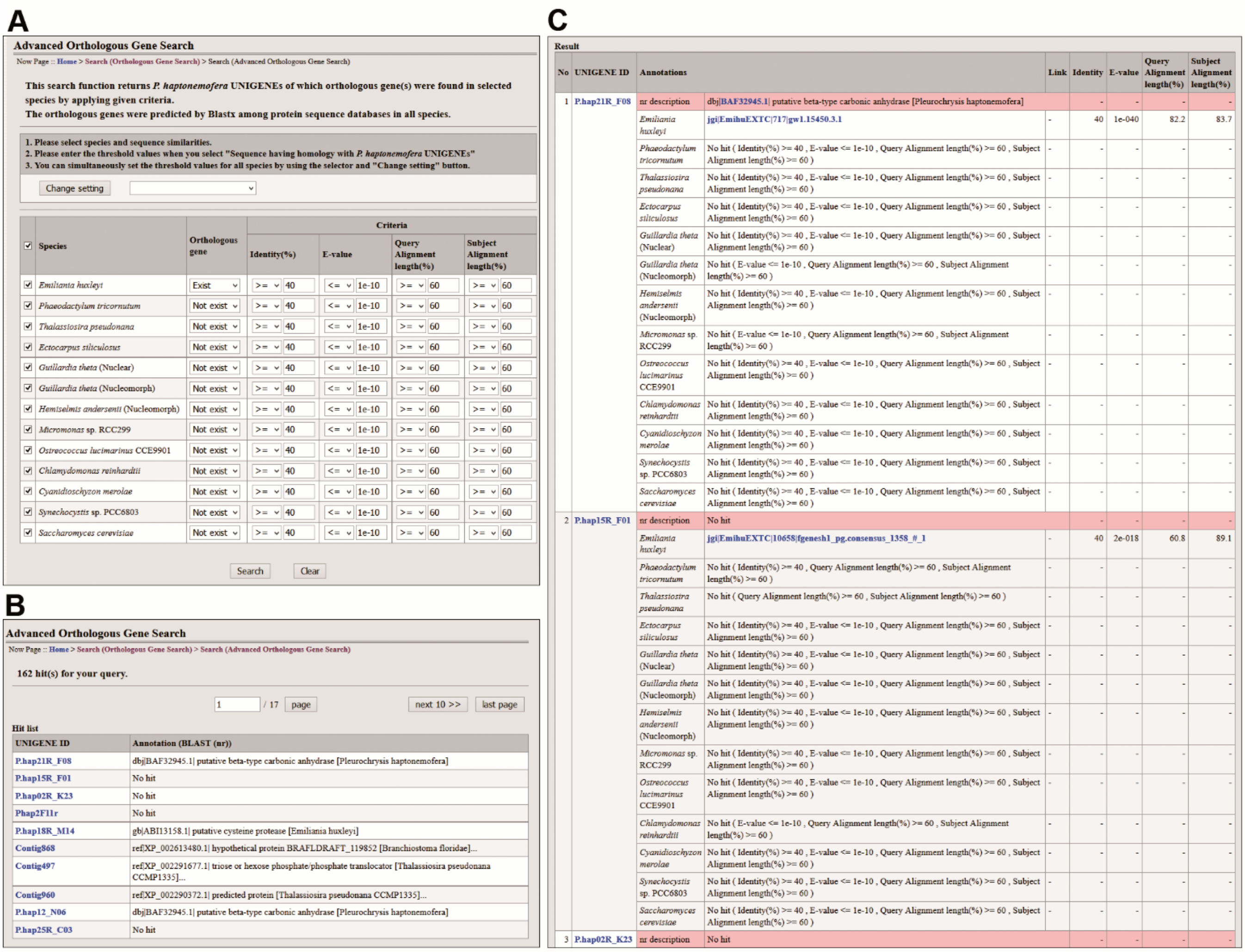

Supplementary Figure S1. A usage example of the 'Advanced Orthologous Gene Search' function. An example of option settings to explore genes conserved in *P. haptonemofera* and *E. huxleyi* but not in other organisms in the 'Advanced Orthologous Gene Search' (**A**), the number of UNIGENEs matched to the search conditions (162 UNIGENEs are matched here) and the 'Hit list' table summarizing the first 10 UNIGENEs including an UNIGENE annotated as a putative beta-type carbonic anhydrase (B), and a part of the 'Result' table showing each *P. haptonemofera* UNIGENE has an orthologous gene only in *E. huxleyi* (C).
